# Coexisting juvenile idiopathic arthritis and autoimmune hepatitis type 1 in a child

**DOI:** 10.1093/rap/rkag101

**Published:** 2026-08-12

**Authors:** Majd Oweidat, Sojoud H Smerat, Fawzy M Abunejma, Jihad Lehroob

**Affiliations:** College of Medicine, Hebron University, Hebron, West Bank, Palestine; Department of Pediatrics, Palestine Red Crescent Specialized Hospital—Hebron, Hebron, West Bank, Palestine; College of Medicine, Hebron University, Hebron, West Bank, Palestine; Department of Pediatrics, Palestine Red Crescent Specialized Hospital—Hebron, Hebron, West Bank, Palestine; Department of Pediatrics, Al-Ahli Hospital, Hebron, West Bank, Palestine; Department of Pediatrics, Palestine Red Crescent Specialized Hospital—Hebron, Hebron, West Bank, Palestine

Key message

- Unexplained transaminase elevation in JIA should prompt evaluation for coexisting autoimmune hepatitis.

Dear Editor, Juvenile idiopathic arthritis (JIA) is the most common chronic rheumatologic disease in childhood, while autoimmune hepatitis type 1 (AIH1) is an immune-mediated liver disease typically associated with ANA and/or anti–smooth muscle antibodies [1, 2].

Herein we describe a 9-year-old girl who presented with 5 weeks of painful left ankle swelling causing limping, morning stiffness and right knee pain following an upper respiratory tract infection. Three weeks earlier, a primary care physician had administered a single intramuscular dose of dexamethasone, which partly improved pain and gait but left persistent ankle swelling and morning stiffness. Perinatal and developmental histories were unremarkable, with no chronic illness, surgery, hepatotoxic drugs, herbal remedies, regular medications or allergies. Several paternal relatives had chronic hepatitis B, but there was no family history of autoimmune or rheumatological disease.

On assessment, she appeared well and afebrile, with normal vital signs and age-appropriate growth. Examination showed a warm, swollen left ankle with mildly reduced range of motion (Fig. 1); other joints were normal, with no rash, oral ulcers, lymphadenopathy, hepatosplenomegaly or ocular abnormalities. Initial tests showed a normal white cell count; mild thrombocytosis; normal inflammatory markers, renal function and albumin; and positive RF (121 IU/ml). Given the recent infection, presentation and partial steroid response, reactive arthritis *vs* JIA was considered and weight-based oral ibuprofen was started.

**Figure 1:**
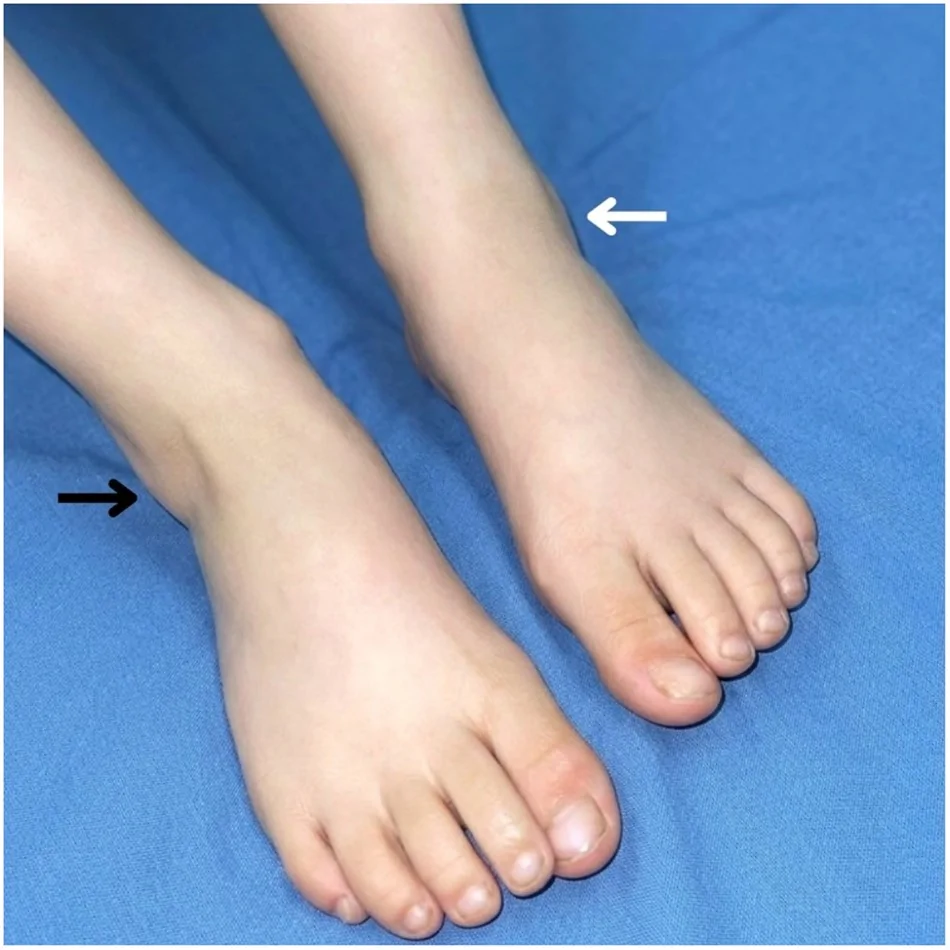
Clinical photograph of the lower extremities showing swelling of the left ankle (white arrow). The right ankle (black arrow) appears normal in comparison

At a follow-up visit 1 week later, ankle swelling and morning stiffness persisted and she developed pain at the left hip and intermittent swelling of the left wrist, without fever, weight loss or gastrointestinal symptoms. Examination confirmed synovitis of the left ankle, pain on movement of the left hip and mild left wrist swelling. After persistence of inflammatory arthritis beyond 6 weeks from symptom onset and exclusion of alternative causes, and under the provisional Pediatric Rheumatology International Trials Organization JIA classification criteria, an inflammatory arthropathy most consistent with chronic unclassified JIA became the leading diagnosis and MTX with folic acid was planned. Anti-cyclic citrullinated peptide antibodies and HLA-B27 were not performed. Routine screening before MTX showed alanine aminotransferase and aspartate aminotransferase around three times the upper limit of normal for her age, with normal albumin, urea, creatinine and coagulation studies. Hepatitis B surface antigen was negative and there was no history of hepatotoxic exposure, so MTX was withheld and she was referred to paediatric gastroenterology.

At gastroenterology assessment, she was asymptomatic from a hepatic or gastrointestinal standpoint; examination showed a soft non-tender abdomen without organomegaly or stigmata of chronic liver disease. Serial liver profiles over the next weeks confirmed persistent transaminase elevation at about two to three times the upper limit of normal with preserved synthetic function and low inflammatory markers. Viral hepatitis screening remained negative. Extensive evaluations for other causes of chronic liver disease, including coeliac disease, inflammatory bowel disease and metabolic conditions, were unremarkable. ANA was initially negative but became positive on repeat testing, whereas anti–smooth muscle and anti–liver-kidney microsomal antibodies remained negative. Serum IgG, IgA and IgM levels were within the reference range.

Because liver enzymes remained persistently abnormal in an otherwise well child with new positive ANA and no evidence of viral or metabolic liver disease, a percutaneous ultrasound (US)-guided liver biopsy was performed. US showed no cirrhosis or focal lesions. Histopathology showed chronic minimally active hepatitis, grade 2, with moderate portal inflammatory infiltrate composed mainly of lymphocytes with few plasma cells and eosinophils, associated with focal lymphoplasmacytic interface hepatitis. Trichrome staining showed periportal fibrosis, grade 1. There was no steatosis, cirrhosis or malignancy. In conjunction with the serological profile and clinical context, these findings were consistent with AIH1.

She was started on oral AZA 1.1–1.2 mg/kg/day and budesonide 6 mg once daily. Disease-modifying therapy for arthritis was deferred until the liver disease stabilized. At joint paediatric gastroenterology and rheumatology follow-up over 2 years the patient remained clinically well, with appropriate growth parameters and good quality of life. She had occasional mild musculoskeletal pain episodes during some months, but these were not associated with signs of inflammation, including joint swelling, tenderness, erythema or limitation of motion. No skin lesions were observed and ophthalmic examinations remained normal. Liver biochemistry, synthetic liver function and other liver-related laboratory parameters remained within normal limits. ESR showed occasional mild fluctuations during follow-up, reaching a maximum of 45 mm/h and subsequently declining. She remains on MTX, AZA maintenance therapy and budesonide tapering is planned soon.

This case highlights the rare coexistence of JIA and AIH1, possibly reflecting shared immunopathogenic mechanisms [3, 4]. Clinicians should consider AIH1 in children with JIA and unexplained transaminase elevation to allow early diagnosis and treatment.

## Data Availability

Data supporting this case report are included in the article.
